# PROTOCOL: Opioid‐specific medication‐assisted therapy and its impact on criminal justice and overdose outcomes

**DOI:** 10.1002/cl2.1138

**Published:** 2021-03-09

**Authors:** C. Clare Strange, Sarah M. Manchak, Jordan M. Hyatt, Cory Haberman, Alisha Desai

**Affiliations:** ^1^ School of Criminal Justice University of Cincinnati Cincinnati Ohio USA; ^2^ Department of Criminology & Justice Studies Drexel University Philadelphia Pennsylvania USA; ^3^ Department of Psychology Drexel University Philadelphia Pennsylvania USA

## Abstract

**Background:**

The overlap between criminal justice system involvement and drug use is well‐documented, and criminal justice agencies have been particularly overwhelmed by the recent opioid epidemic. Treating opioid (and other substance) addiction as a means to reduce risk for future criminality and improve public safety is inherently a responsibility for the criminal justice system. In turn, the criminal justice system has a responsibility to manage and treat addiction among the individuals under its purview.  Policy recommendations place emphasis on the use of medication‐assisted treatments (MAT) as a front‐line defense among correctional populations, because its efficacy and effectiveness has been well‐established in other contexts.  Despite this, criminal justice agencies have been reluctant or slow to do so.

**Objectives:**

The current review will provide criminal justice and substance use treatment decision‐makers with information regarding the efficacy and effectiveness of opioid‐specific MAT on offending and overdose outcomes. Specifically, the authors will address the following research questions: Do opioid‐specific MATs reduce the frequency or likelihood of criminal justice outcomes, as defined by official or self‐reported indices of criminal reconviction or rearrest, revocation of community supervision, mandated treatment failure, and specialized court docket failure? Do opioid‐specific MATs reduce the frequency of opioid overdose among individuals with current or prior self‐reported or official record of criminal justice system involvement?

**Inclusion Criteria:**

Studies were required to use strong quasi‐experimental or randomized experimental designs. All studies used individual level unit of analysis and examined adults and adolescents who are male, female, or nonbinary and racially/ethnically diverse, with current opioid use and who have current or prior criminal justice involvement. Studies had to prospectively test the effects of heroin and methadone maintenance, buprenorphine, or naltrexone on criminal conviction, arrest, revocation of community supervision, technical probation or parole violation, mandated treatment failure, and specialized court docket failure. Overdose outcomes were also examined for samples in criminal justice settings such as jails, prisons, probation, and parole.

**Search Strategy and Data Collection:**

This review builds upon a prior review conducted by Egli et al. (2009) and examined studies meeting the inclusion criteria above published between 1960 and October 31, 2020. The following platforms and databases (in parentheticals) were used: EBSCOhost (Criminal Justice Abstracts, SocINDEX with Full Text, Legal Collection, Wilson Omnifile, PsycINFO, Social Work Abstracts, and Women's Studies International [includes grey literature]); ProQuest (Criminal Justice Database, PAIS [includes grey literature], Dissertations and Theses Global [includes grey literature]); Gale (Expanded Academic ASAP, Opposing Viewpoints Resource Center); FirstSearch (GPO Monthly Catalog, PapersFirst [includes grey literature]); ISI Web of Knowledge (Web of Science Core Collection); Office of Justice Programs (National Criminal Justice Reference Service); Summon; and Nexis Uni. The following open access platforms and databases will also be consulted: Elsevier (Scopus [includes grey literature]); Science.gov; ClinicalTrials.gov; WHO International Clinical Trials Registry Platform (ICTRP) portal; and Google Scholar. Search terms were harvested according to their demonstrated success in drawing out relevant and complete results for studies regarding the effectiveness of opioid‐specific medication‐assisted therapies (MATs). From this process 5 core search strings were created, each one with the same general base terms, but unique outcome measure(s).

**Analysis:**

For binary offending outcomes (e.g., arrest, conviction, incarceration, specialty court failure, mandated treatment failure, or community supervision failure) and overdose outcomes, odds ratios were computed, and for continuous or quasi‐continuous outcomes (e.g., total number of arrests), a standardized mean difference type effect size was computed and then transformed into an odds ratio. We used the *χ* ^2^ test that goes with the forest plot and computed the *I* ^2^ statistic to assess heterogeneity. Risk of bias was assessed with (1) the revised Cochrane risk‐of‐bias tool for randomized trials; and (2) the risk of bias in non‐randomized studies of interventions assessment tool.

## 1 BACKGROUND

### 1.1 Problem, condition, or issue

Opioids have become increasingly available worldwide and pose some of the most serious health consequences, as compared to other types of drugs (United Nations Office on Drugs & Crime, 2015). In 2018, 58 million people used opioids worldwide. Over two‐thirds of deaths due to drug use are from opioids, and in 2017, over 115,000 died from an opioid overdose (World Health Organization [WHO], 2020).

The overlap between criminal justice system involvement and drug use is well documented across a variety of countries and samples (e.g., Boutwell et al., 2007; Dolan et al., 2007; European Monitoring Centre on Drugs and Drug Addiction [EMCDDA], 2012; Winkelman et al., 2018). Once released from secure correctional facilities, people with opioid addiction are at high risk for relapse and criminal recidivism. Specifically, a meaningful minority of deaths of former inmates is attributable to opioid overdose (Binswanger et al., 2013; Singleton et al., 2016; World Health Organization [WHO], 2010), and a significant percentage of former inmates will recidivate within 5 years (Fazel & Wolf, 2015).

Criminal justice agencies have been particularly overwhelmed by the recent opioid epidemic. Treating opioid (and other substance) addiction as a means to reduce the risk for future criminality and improve public safety is inherently a responsibility for the criminal justice system, as the influence of substance use on criminal activity is well documented in the literature (Bonta & Andrews, 2017). Of course, one could also argue that opioid addiction, its withdrawal symptoms, and its recovery constitute a serious medical condition for which criminal justice agencies have a responsibility to treat and manage in accordance with the United Nations requirements for the Basic Principles for the Treatment of Prisoners (United Nations, 1990). In fulfilling their responsibility to provide adequate healthcare for individuals in their custody, and through their efforts to rehabilitate offenders to reduce their risk for recidivism more generally, correctional providers must treat substance use disorder. When they do, they may impact criminal recidivism as well as health outcomes like future overdose. As such, it is necessary to deploy the most effective treatment available to achieve maximum impact on these outcomes.

Policy recommendations (WHO, 2009) place emphasis on the use of medication‐assisted treatments (MAT) as a front‐line defense among correctional populations, because its efficacy and effectiveness have been well established in other contexts (Belenko et al., 2013; Koehler et al., 2014). Despite these policy recommendations, criminal justice agencies have been reluctant or slow to do so (Friedmann et al., 2012; Matusow et al., 2013; Parrino et al., 2015). Many factors may contribute to the poor uptake of this particular approach for managing and treating opioid addiction. It is possible that practitioners may question the utility for MAT to impact public safety outcomes—the chief policy concern of the criminal justice system. Indeed, the uptake of psychological research evidence—particularly that which establishes a strong link between addiction and criminality—into correctional policy and practice has been slow, at best (Gannon & Ward, 2014). Moreover, there may be confusion or even hesitation among practitioners in correctional settings about their responsibility to encourage or administer an intervention that traditionally falls under the purview of healthcare providers.

### 1.2 The intervention

There are a variety of MAT drugs that are currently used for the management and treatment of opioid addiction. This review focuses on those most modern and commonly used drugs to treat opioid addiction over the long term, in the form of supervised maintenance programs, drug substitution, or antagonist protocols. Thus, this review will not examine the effects of Naloxone, which is a drug used to revive someone in a singular emergent opioid overdose event.

The drugs examined in the current review include opioid agonists (heroin and methadone), partial agonists (buprenorphine), and antagonists (naltrexone). Opioid agonists are drugs that work on the opioid receptors in the brain and produce a full opioid effect. Heroin and methadone maintenance MAT services must be administered under the supervision of medical professionals in a highly controlled environment and on a regimented schedule. This approach is designed to help reduce the illicit or off‐label use of opioids, cravings, and, gradually, the amount of opioid intake over time. Partial agonists such as buprenorphine also operate on the opioid receptors but produce weaker euphoric effects than felt with full agonists. This class of MAT is also designed to help lower dependency symptoms, misuse, cravings, and symptoms of withdrawal. Buprenorphine is a longer acting agent, so it can be administered less frequently and has the approval to be administered in a variety of clinical settings. Opioid antagonists like naltrexone block the opioid receptors entirely so that if a patient used an opioid, he or she will not be able to achieve any euphoric effects. It is designed to relieve withdrawal and cravings and must be administered by a doctor, nurse, or nurse practitioner.

### 1.3 How the intervention might work

The impact of opioid‐specific MAT on overdose outcomes is well established. MAT reduces cravings, illicit drug use, and the amount of opioid use over time (Belenko et al., 2013; Koehler et al., 2014). All of these, in turn, aid to the reduction of overdose outcomes. The mechanisms by which opioid‐specific MAT impact criminal justice outcomes are less understood. However, prior research on substance use treatment in general and correctional rehabilitation theory suggests MAT could reduce criminal risk. As substance use is a robust predictor of criminal involvement, reducing substance use reduces future criminal involvement (see Bonta & Andrews, 2017). By extension, any intervention targeting addiction, including MAT, should operate to reduce recidivism risk. More specifically, because MAT facilitates reductions in risky drug use, opioid users will engage less frequently or not at all in drug‐related behaviors that warrant a criminal justice response (i.e., drug use, possession, trafficking, paraphernalia possession). Similarly, by reducing cravings and use, people will no longer be motivated to engage in criminal activity that supports, or fuels, their addiction (e.g., burglary).

### 1.4 Why it is important to do this review

The current evidence base for MAT on reoverdose outcomes is strong, but little is known about its impact on the subsample of people with an opioid addiction who also come into contact with the criminal justice system. Because these individuals face challenges posed by addiction and criminal justice involvement, they likely have different experiences and thus needs and risks than people not facing this combination of challenges. Thus, it is necessary to identify whether the same positive clinical outcomes seen among nonoffender groups can be observed among people with criminal justice involvement. Further, although addressing substance use should—and does—reduce criminal risk (Bonta & Andrews, 2017), it is unclear if it is enough to reduce recidivism for people with a serious opioid addiction. A rigorous and systematic synthesis of the evidence base on the effectiveness of MAT for improving public safety will allow criminal justice agencies to make informed decisions about policy, practice, and the allocation of resources. In light of the range of MAT options currently available and the pressing need for methodologically robust results and changes to the underling legal and public health landscape, an updated and complete review is particularly policy‐relevant today.

This systematic review will be an update and modification of a 2009 Campbell Systematic Review entitled “Effects of Drug Substitution Programs on Offending among Drug‐Addicts” (Egli et al., 2009). Although the authors of this review reported the intent to publish an update every 5 years, no update has yet been published. To the current authors' knowledge, an update is also not currently in progress or planned. As 10 years of research has amassed on this topic, particularly during the height of the “opioid epidemic,” it is necessary to update this review. Further, this review will be far more comprehensive in focus than any reviews on the topic performed to date, because it will include multiple outcomes observed among criminal justice samples and incorporate studies on a variety of pharmacological interventions for opioid use—some of which are quite new.

## 2 OBJECTIVES

The current review will provide criminal justice and substance use treatment decision‐makers with information regarding the efficacy and effectiveness of opioid‐specific MAT on offending and overdose outcomes. Specifically, the authors will address the following research questions.

1. Do opioid‐specific MATs reduce the frequency or likelihood of criminal justice outcomes, as defined by official or self‐reported indices of criminal reconviction or rearrest, revocation of community supervision, mandated treatment failure, and specialized court docket failure?
2. Do opioid‐specific MATs reduce the frequency of opioid overdose among individuals with a current or prior self‐reported or official record of criminal justice system involvement?

The answers to these research questions will help to inform criminal justice and substance use treatment policymakers on the usefulness of opioid‐specific MATs in reducing criminal justice outcomes in criminal justice settings, or overdose outcomes in treatment settings with the criminal justice population. In order to prevent the biased or incomplete summary of the evidence, the current review will also highlight any reported adverse effects of the implementation of opioid‐specific MATs with criminal justice‐involved populations. Additionally, it will be important to consider the potentially variant impact of MAT on multiple subgroups of criminal justice‐involved people (e.g., racial, ethnic, gender, age, socioeconomic), as will be addressed through the analysis of effect moderators (see Section 4.10). Implementing or maintaining an opioid‐specific MAT program is a practical decision that requires the use of program resources, and therefore it is important that program leadership have a foundational understanding of the intervention and its established efficacy and effectiveness.

## 3 METHODS

The current review will be a Campbell Collaboration update to, and expansion of “Effects of Drug Substitution Programs on Offending among Drug‐Addicts” (Egli et al., 2009). The original review analyzed the impact of drug substitution therapies on criminal offending. To maintain the integrity of the update, the current review will employ a similar method to the original study, including certain search strategies and databases, criteria for inclusion, and coding and statistical procedures. As such, the method described subsequently is similar to the method described in Egli et al. (2009) with some small alterations.

### 3.1 Criteria for considering studies for this review

#### 3.1.1 Types of studies

To be eligible for inclusion in this review, studies are required to use strong quasi‐experimental or randomized experimental designs that prospectively test the effects of the MAT for opioid use disorder on criminal and overdose outcomes. Due to the difficulty of conducting randomized controlled trials (RCTs) in criminal justice settings, it is necessary to examine quasi‐experimental studies that employ more rigorous design features. Specifically, all quasi‐experimental studies are required to either use a matching procedure when testing differences in the treatment and comparison groups or use statistical controls for baseline group differences. All studies are required to use an individual‐level unit of analysis.

#### 3.1.2 Types of participants

The considered population will consist of opioid‐using adults and adolescents who are male, female, or nonbinary and racially/ethnically diverse. All participants must have current opioid use, as indicated by self‐report or diagnosis; participants will not be required to have an opioid‐specific substance use disorder but are likely to, given MAT is typically administered for people with this diagnosis only. Additionally, all participants must have current or prior criminal justice involvement, as indicated by the self‐report or official report of the prior or current arrest, incarceration, charges, or convictions.

#### 3.1.3 Type of interventions

In contrast to the original review, which included MAT treatment for other illicit substance use (e.g., cocaine), this review focuses only on MAT for opioid use disorder. Specifically, this review includes studies that test the impact of heroin and methadone maintenance, buprenorphine, or naltrexone as the independent variable. The comparison and control group for the quasi‐experimental and experimental designs, respectively, may be any intervention that is not an opioid‐specific MAT (i.e., alternative medication not specifically intended for opioid use treatment [e.g., antidepressant]), “talk therapy” (i.e., any individual or group counseling, using any theoretical model or approach; e.g., cognitive‐behavioral therapy, group processing, psychotherapy), no intervention, or a placebo. Additionally, the review will also allow for a comparison of two opioid‐specific MAT conditions (e.g., methadone vs. buprenorphine), as well as combined MAT + talk therapy versus a comparison condition fitting the above criteria. We will not impose restrictions on the number of treatment versus control conditions we may encounter. Because research, particularly biomedical or pharmaceutical research, with criminal justice populations can be logistically challenging, we do not anticipate many studies with multiple conditions.

The current review will include studies of opioid‐specific MAT meeting the inclusion criteria, regardless of where it is administered or delivered (community, court, or institutional). This is a modification of the original review, which excluded incarceration‐based treatment programs. Because the current review's outcomes will extend beyond such criminal justice measures as reconviction, incarceration‐based programs remain appropriate for inclusion.

#### 3.1.4 Types of outcomes

The primary dependent variable, criminal justice involvement, can be determined through self‐report or official record, and may include any of the following outcomes: conviction, arrest, revocation of community supervision, technical probation or parole violation, mandated treatment failure, and specialized court docket failure. The full list of outcomes represents a modification of the original review, which included only crime‐related outcomes defined as: earning from illegal activity, self‐reported, or official indices of the commission of a new crime, police contact, arrest, reconviction, probation violation, or incarceration, and the cost of crime.

The secondary dependent variable examined is opioid overdose, which can also be determined through self‐report or official record. However, only studies that employ a sample of, or have analyses specific to, people with current or prior criminal justice involvement, as indicated from self‐report or official record, will be included. In other words, this review will not include studies that examine overdose outcomes for people who have no current or prior criminal justice involvement or if this information is missing from the manuscript.

### 3.2 Search methods for identification of studies

#### 3.2.1 Electronic searches

The original review included relevant studies identified through databases such as Campbell Crime and Justice Group, National Criminal Justice Reference Service, MEDLINE, National Treatment Agency for Substance Misuse, National Treatment Outcome Research Study, Central Committee on the Treatment of Heroin Addicts, Criminal Justice Abstracts, and JSTOR. The current review will include all studies from Egli et al.'s (2009) review, in addition to studies independently selected by the authors of the current review that were published from 1960 to October 31, 2020. Thus, any studies published on or after November 1, 2020, are not included in this review.

The studies for the current review will be accessed on the following platforms (via access from the University of Cincinnati), followed by the specific databases in parentheticals: EBSCOhost (Criminal Justice Abstracts, SocINDEX with Full Text, Legal Collection, Wilson Omnifile, PsycINFO, Social Work Abstracts, and Women's Studies International [includes gray literature]); ProQuest (Criminal Justice Database, PAIS [includes gray literature], Dissertations and Theses Global [includes gray literature]); Gale (Expanded Academic ASAP, Opposing Viewpoints Resource Center); FirstSearch (GPO Monthly Catalog, PapersFirst [includes gray literature]); ISI Web of Knowledge (Web of Science Core Collection); Office of Justice Programs (National Criminal Justice Reference Service); Summon; and Nexis Uni. The following open access platforms and databases will also be consulted: Elsevier (Scopus [includes gray literature]); Science.gov; ClinicalTrials.gov; WHO International Clinical Trials Registry Platform (ICTRP) portal; and Google Scholar.^1^ These databases are identified as common databases used in criminal justice research, at the expertise of several general and criminal justice‐specific librarians at the University of Cincinnati, and include such important unpublished sources as conference papers and gray literature.

#### 3.2.2 Searching other sources

Furthermore, and similar to Egli et al. (2009), the authors of the current review will consult the bibliographies of other relevant reviews for additional studies to include, as well as the bibliographies of the included studies themselves. Irrespective of electronic availability, the authors will contact university libraries and first/corresponding authors in order to retrieve all articles that appear to meet the criteria for inclusion. Lastly, to reduce publication bias, the authors of the current review will also contact the authors of the included studies for any unpublished data or manuscripts that reported results, including dissertation studies.

For the current review, search terms were harvested according to their demonstrated success in drawing out relevant and complete results for studies regarding the effectiveness of opioid‐specific MATs. This method was adapted from the rigorous strategies often employed in systematic reviews from the medical field. First, 10 “gold‐standard” articles were selected from Egli et al.'s (2009) review (i.e., those studies that best reflected the type of studies desired for the current review, both methodologically and in subject matter). These articles were entered into the PubMed database, where a Medical Subject Heading (MeSH) analysis generated a list of common terms across all ten gold‐standard articles (see Appendix C for full MeSH analysis). The research team identified relevant terms from the MeSH analysis and then brainstormed potential variants of each term and Boolean operators (including variants of the terminology, spelling, use of quotations, and truncations) in order to determine the version of each term that was most likely to draw complete and relevant results. Each term was tested using the Criminal Justice Abstracts database for its breadth of subject matter. From this process, five core search strings were created, each one with the same general base terms, but unique outcome measure(s). Search strings were created such that studies were retrieved if they contained any of the base terms, and the outcome listed in Appendix D.

As needed in order to draw out more relevant search results, the base of each search string will be altered according to the type of database being searched (e.g., medical vs. criminal justice literature) and the functionality of the database's search function. All final search strings and their modifications will be recorded and reported in the final review for the purposes of study replication. Each search string will be entered into each database, and the total number of studies retrieved from each string will be recorded (after duplicates are removed). Results from all source types will be considered in the initial phase of the search (e.g., newspapers, journals, letters, conference abstracts).

## 4 DATA COLLECTION AND ANALYSIS

### 4.1 Description of methods used in primary research

The studies to be included in the current review will employ an experimental or strong quasi‐experimental design examining the impact of specified MATs on individual‐level criminal and overdose outcomes. All studies will include a measure of the impact of an opioid‐specific MAT on offending or overdose outcomes within criminal justice‐involved populations.

In the quasi‐experimental and experimental studies, the treatment group will receive an opioid‐specific MAT (e.g., Buprenorphine, naltrexone, methadone maintenance, heroin maintenance), and the control group may receive a different type of opioid‐specific MAT (e.g., methadone compared to Buprenorphine), a placebo, some sort of alternative medication not specific to opioid addiction, talk therapy(e.g., individual or group counseling), or no treatment at all. Additionally, the treatment condition may also be a MAT + talk therapy treatment. All talk therapy interventions will be further coded into cognitive‐behavioral (CBT) versus other, since cognitive‐behavioral therapies traditionally produce greater effects and have a larger evidence base than other approaches in the treatment of substance use disorder (McHugh et al., 2010).

### 4.2 Criteria for determination of independent findings

As discussed in Egli et al. (2009), there are three potential avenues for the nonindependence of findings: (a) multiple indicators of offending reported from a single study (e.g. arrest, conviction); (b) the same outcome measured at multiple points in time; and (c) the same data being reported across multiple studies (e.g., in multiple past systematic reviews). The criteria for the determination of independent findings will be the same for the current review as was outlined in the Campbell Review Protocols (e.g., Lipsey & Landenberger, 2006).

In the event that there are multiple indicators of offending reported from a single study, the authors will select a commonly used outcome measure from other studies, the “all offenses” category, typically using a measure of rearrest. In the event that the “all offenses” category is not available, an offense category will be selected at random (see Egli et al., 2009; Lipsey & Landenberger, 2006). This will be done in an effort to maintain as much comparability across studies as possible.

In the event that the same outcome is measured at multiple points in time, the authors will include the measure which has the closest timing to those most commonly used across the other studies (i.e., outcome at 12 months). This is again to encourage as much comparability as possible given the unique methods employed across some studies (Lipsey & Landenberger, 2006).

Lastly, in the event that the same data are reported across multiple studies, the coding protocol will include measures that allowed the current review authors to identify the shared reporting of data. This will include identifiers of each study's authors' names, location, and time frame of the study, as well as sample descriptions. In the event that multiple publications report results using the same set of data, the study with the most complete and detailed manuscript will be used as the primary coding source. From there, the coding protocol will also track which manuscript will be the primary coding source, and whether there are other manuscripts reporting the same data. These additional manuscripts will only be consulted given the need for clarification surrounding the data in the primary designated manuscript.

### 4.3 Selection of studies

The research team will review all potentially relevant studies for the proper inclusion criteria using the same coding system as the original study, made available to the current authors by Egli et al.'s (2009) team. All studies will be coded in two phases. In the first phase, the titles and abstracts will be reviewed to determine basic inclusion criteria is met—that is, (a) the evaluation of effectiveness of MAT services (b) on criminal or overdose outcomes (c) for people with opioid use disorder (d) who are or have been involved in the criminal justice system. Studies meeting these criteria or any study for which this information cannot be readily determined from the title or abstract will be retained for coding in Phase 2. In Phase 2, the full text of each study will be reviewed. All studies with irrelevant independent or dependent variables, ineligible sample characteristics, and studies that lack the methodological rigor will be removed from consideration for inclusion. The number of studies removed during this process will be recorded and reported.

To ensure reliability regarding exclusion, the lead and corresponding authors will each independently screen half of the pool of potential studies for inclusion. If at any time, one coder is unsure about a study's eligibility for inclusion (e.g., criteria for inclusion are not detailed enough or are too vague to render a decision), the coder will render a preliminary decision and ask the other coder to review and code the article independently as a “cross‐check” on reliability. In the event that the lead and corresponding author cannot reach consensus, the third author will make the determination. The number and outcome of these reliability cross‐checks will be recorded and reported. As a final failsafe, each coder will also independently code a random sample of 5% of the total studies included in the other coder's group, and appropriate reliability coefficients for agreement (kappa for categorical variables and ICC for continuous variables) will be computed. In the event that the prespecified eligibility criteria are adjusted by the team according to unanticipated issues that may arise during the search or coding stages, the team will document these suggested changes (in place of making ad hoc decisions about inclusion that stray from the protocol) and perform sensitivity analyses with and without these studies that may no longer meet the eligibility criteria.

### 4.4 Data extraction and management

The Egli et al. (2009) team created a coding protocol for the original review that provided for a systematic method of extracting information regarding each study's research design, program, nature of the outcome measures, and outcome data. This protocol was provided to the current research team in order to promote consistency and structure in the coding procedures used in the updated review. The current research team then updated this coding protocol to reflect the changes from the original to the current review. The updated coding protocol includes the systematic extraction of information regarding the study identification, content and methodological inclusion criteria and rigor, control and treatment sample descriptive information, actions taken upon the control and treatment samples, treatment characteristics, the types and measurement of outcome data, and effect size information. See Appendix A for the updated coding protocol. Coding reliability will be established through the use of multiple coders (i.e., at least two for any given study) to independently code each study. Discrepancies in coding will be brought to an additional coder to make the final coding decision.

### 4.5 Assessment of risk of bias in included studies

We will use two tools to assess risk‐of‐bias in our included studies: (a) The Revised Cochrane risk‐of‐bias tool for randomized trials (RoB 2); and (b) The Risk of Bias in Nonrandomized Studies−of Interventions (ROBINS‐I) assessment tool.

The necessary steps will be taken to assess the risk of bias in randomized trials using the RoB 2. First, as part of the preliminary considerations two independent coders will document the important characteristics of each study and the sources that are used to confirm these characteristics using the standard template provided in the tool. Then, using the appropriate version of the RoB 2 (given the type of randomized trial), signaling questions, criteria, response options, and scoring algorithm for each domain we will determine which studies are at “low risk of bias,” “high risk of bias,” or present “some concerns of bias.” If at least one domain is determined to present “some concerns” or “high risk of bias,” then the study will maintain this risk‐of‐bias rating overall. All domains will be assessed for each study even after one domain may be rated as “some concern” or “high risk of bias.” The domains include: (a) bias arising from the randomization process; (b) bias due to deviations from intended interventions; (c) bias due to missing outcome data; (d) bias in measurement of the outcome; and (e) bias in selection of the reported result. Additionally, cluster‐randomized trials will be assessed for bias arising from identification or recruitment of individual participants within clusters. Judgments will be overridden only in the event that the full authorship team is in agreement that it is appropriate. Domain‐level consensus judgments will be displayed in a table in the main document and the answers and justifications for each signaling question will be provided in an appendix.

The ROBINS‐I tool will be used to assess risk‐of‐bias in nonrandomized studies of the effects of interventions (NSRIs) and will follow the same general process as the RoB 2 described above, but with necessary emphasis on content expertise. In preparation for assessing risk‐of‐bias within NSRIs, a preliminary specification of the anticipated key confounders and cointerventions for each study will be identified in consideration of the review's essential research questions. Target trials (i.e., hypothetical randomized trials whose results mimic those of the specific NSRI) will also be specified. Then, using the ROBINS‐I signaling questions, criteria, response options, and scoring algorithms we will assess risk‐of‐bias across its seven domains: (a) bias due to confounding; (b) bias in the selection of participants into the study; (c) bias in classification of interventions; (d) bias due to deviations from intended interventions; (e) bias due to missing data; (f) bias in the measurement of outcomes; and (g) bias in the selection of the reported result. Each domain will be rated one of the following: “low risk of bias,” “moderate risk of bias,” “serious risk of bias,” “critical risk of bias,” and “no information,” with accompanying text for justification. Studies will assume the overall risk level of the “riskiest” domain. Attempts will be made to determine the magnitude and potential direction of biases. Upon all determinations, the studies’ overall strengths and weaknesses will be compared and contrasted to determine the degree to which intervention effects might be considered causal. As with the RoB 2, all domain‐level consensus judgments will be displayed in a table in the main document and the answers and justifications for each signaling question will be provided in an appendix.

### 4.6 Measures of treatment effect

The statistical procedures and conventions will align closely with those that were used in Egli et al.'s (2009) review, as the types of studies and outcomes that were included will be similar. The most detailed numerical data will be collected in order to facilitate similar analyses across the included studies. For binary offending outcomes (e.g., arrest, conviction, incarceration, specialty court failure, mandated treatment failure, or community supervision failure) and overdose outcomes, odds ratios will be computed, which are typical and well‐suited for dichotomous outcomes. For continuous or quasi‐continuous measures of these outcomes (e.g., total number of arrests), a standardized mean difference type effect size will be computed, and then transformed into an odds ratio (Egli et al., 2009; Lipsey & Wilson, 2001).

In multiarm studies, in which there is more than one comparator condition and the additional comparator conditions are determined to be eligible for this systematic review, researchers will attempt to combine intervention and comparator conditions so that only a single pairwise comparison is computed, in line with recommendations from Higgins et al. (2019). This prevents an intervention group from being “double‐counted” and inflating the unit of analysis error (Higgins et al., 2019). Also, consistent with MECCIR Conduct Standards, all intervention groups, regardless of inclusion, will be detailed in the Section 3.1 (Higgins et al., 2019).

### 4.7 Dealing with missing data

It is possible that missing data from the original studies can preclude either (a) sound decision‐making concerning study eligibility and selection for inclusion and/or (b) ability to compute an effect size. In both instances, the researchers will attempt to obtain the relevant data necessary from the original study authors. If this is not possible, the researchers will include the studies in the summary of studies in a narrative form and will detail why they were not included in the meta‐analysis.

### 4.8 Assessment of heterogeneity

To assess heterogeneity, we will use the *χ* ^2^ test that goes with the forest plot. A *p* value of .10 will be set as the cutoff, because higher quality studies are likely to have smaller sample sizes. Consistent with Cochrane analytic guidelines, we will also compute the *I* ^2^ statistic, which “describes the percentage of the variability in effect estimates that is due to heterogeneity rather than sampling error (chance)” (Deeks et al., 2019).

### 4.9 Data synthesis

The current review will comply with the standards of meta‐analysis as specified in *Practical Meta‐Analysis* by Lipsey and Wilson (2001) and will use SPSS for the analyses. The two types of studies to be included (RCTs and quasi‐experiments) will be meta‐analyzed together using meta‐analysis software. In the event that studies include multiple treatment arms, we will combine eligible multiple treatment versus comparator conditions so that there is only one pairwise comparison made, in the manner detailed below:

1. Synthesis for MAT v placebo/no treatment/wait­list control.
2. Synthesis for MAT v non­MAT pharmacological intervention (e.g., antidepressant).
3. Synthesis for MAT v psychological therapy individual.
4. Synthesis for MAT v psychological therapy group.
5. Synthesis MAT + psychological therapy individual v comparison 1.
6. Synthesis MAT + psychological therapy individual v comparison 2.
7. Synthesis MAT + psychological therapy individual v comparison 4.
8. Synthesis MAT + psychological therapy group v comparison 1.
9. Synthesis MAT + psychological therapy group v comparison 2.
10. Synthesis MAT + psychological therapy group v comparison 3.
11. Synthesis for MAT 1 v MAT 2 (or another MAT treatment­treatment comparisons).

In line with Egli et al. (2009), effect size outliers (considered greater than or equal to 3 standard deviations above or below the mean) will be winsorized to the next highest observation that is not judged to be an outlier. This will prevent bias from extreme observations from entering the results. No data will be imputed for missing values.

In order to compute mean‐effect sizes, the inverse variance weight method of the meta‐analysis will be used (Lipsey & Wilson, 2001). A random‐effect model will be assumed a priori.

### 4.10 Subgroup analysis and investigation of heterogeneity

The a priori determined potential moderators of the effectiveness of opioid‐specific MATs on criminal justice and overdose outcomes that will be explored include design type (experimental or quasi‐experimental), whether or not the opioid‐specific MAT prescription was coupled with talk therapy (e.g., CBT or non‐CBT individual or group counseling), the level of medication adherence, dosage, follow‐up period, and/or treatment length. Each of these potential moderators may contribute to or subtract from the “ideal” treatment conditions as experienced by study subjects. For example, MATs may have a stronger impact over longer periods of time as subjects learn to manage side effects and get into a routine of adherence. In this scenario, studies with shorter follow‐up periods may not capture the full impact of MAT on criminal justice and overdose outcomes.

Other potential moderation analyses will be conducted using metaregression techniques (e.g., Deeks et al., 2019) to examine effects across specific subgroups based on demographics characteristics of gender, race, and age, as well as location/context of treatment (e.g., jail, prison, community, court), and “era” (i.e., did the treatment occur before or after the onset of the opioid crisis). These secondary potential moderators capture the nuances with which criminal justice or overdose outcomes under MAT may vary. For example, MAT may be more effective when implemented in jail versus the community, as administering the medication is in that context the responsibility of the correctional staff, and not the subject.

In the incident where clustering is present in a given study—that is, when multiple cohorts are included in the same treatment arm—we will make adjustments consistent with Cochrane recommendations (Higgins et al., 2019).

### 4.11 Sensitivity analysis

Sensitivity analyses will be conducted post hoc in order to test the robustness of the results. To this end, we will use metaregression to directly examine the impact of small versus large sample sizes (as recommended by Deeks et al. (2019).

## CONFLICT OF INTERESTS

One member of the author team, Jordan M. Hyatt, has conducted a study that could potentially be included in this review. The team will take the steps to ensure that he is not involved in the decision‐making concerning its inclusion or exclusion. The other authors declare that there are no conflict of interests.

## Footnotes

1 The URLs and search fields for these sites are included in Appendix B.

## APPENDIX A CODING PROTOCOL

2. **ELIGIBILITY CRITERIA**

A. **Content**

To be eligible a study must meet one of the following criteria. Answer each question with a “yes” or “no.”

**Table:**

	YES	NO
The study evaluates the effects of MAT on the offending outcomes (e.g., criminal reconviction or rearrest, revocation of community supervision, mandated treatment failure, and specialized court docket failure). The measure may be based on official records or self‐report and may be reported on a dichotomous or continuous scale among opioid users:		
– Comparing delinquency rates among subjects of an experimental group before, during, and following treatment to those of a control group (with or without random assignment).
The study reports a measure of opioid overdose for a criminal justice‐involved population receiving MAT.		

Notes:
B.
**Assignment to groups:**

**Inclusion:** only studies meeting criteria a, b, c, or d will be included

**Table:**

	YES
a. Randomized	
b. Quasi‐randomized	
c. Matched groups	
d. Use of control variables to account for initial group differences which go beyond sex, age, previous convictions/offenses, and offense type	
e. Use of control variables limited to sex, age, previous convictions/offenses, and offense type	
f. No use of control variables	

4. **METHODOLOGICAL RIGOR ASSESSMENT**

**Table:**

A. **Used control variables in statistical analysis to account for initial group differences (yes = 1; no=0).**	**[cntrvar] ___**
B. **Used subject‐level matching (yes = 1; no=0)**	**[matching] ___**
C. **Variables used to control/match on pre‐test differences**	

	**YES**
a. age at the beginning of the program	
b. gender	
c. marital status	
d. education, employment, or economic status	
e. ethnic background or national origin	
f. criminal story:	
Age at first detention	
Prior records	
Type of offenses	
Number of drug‐related charges	
g. Addiction story:	
Age at first consumption	
Type of drugs	
Previous treatments	
h. other:	

C. **Variables used for statistical control**

**Table:**

	**YES**
a. age at the beginning of the program	
b. gender	
c. marital status	
d. education, employment, or economic status	
e. ethnic background or national origin	
f. criminal story:	
Age at first detention	
Prior records	
Type of offenses	
Number of drug‐related charges	
g. Addiction story:	
Age at first consumption	
Type of drugs	
Previous treatments	
h. other:	

D. **Rating of initial group similarity:**	**[simRate] ___**
**(7 highly similar; 1= highly dissimilar)**	
7‐ Randomized design, large N or small N with matching;	
5‐ Nonrandomized design with strong evidence of initial equivalence;	
1‐ Nonrandomized design, comparison group highly likely to be different from the treatment group or known differences that are related to future reoffending	

A. **Attrition problems have been a problem: (yes** = **1; no** = **0)**	**[attrit] ___**

B. **Use of statistical significance test: (yes** = **1; no** = **0)**	**[SigTest] ___**

## APPENDIX B SEARCH PLATFORMS, DATABASES, URLs, AND SEARCH FIELDS

**Table:**

Platform	Database	URL	Search field
EBSCOhost	Criminal Justice Abstracts	http://web.a.ebscohost.com.proxy.libraries.uc.edu/ehost/search/advanced?vid=0%26sid=020904cf-79ab-4414-a7c2-75d3bb035837%40sessionmgr4008	“TX ALL Text”
EBSCOhost	SocINDEX with Full Text	http://web.a.ebscohost.com.proxy.libraries.uc.edu/ehost/search/advanced?vid=0%26sid=020904cf-79ab-4414-a7c2-75d3bb035837%40sessionmgr4008; then “Choose Databases” > SocINDEX with Full Text	“TX ALL Text”
EBSCOhost	Legal Collection	http://web.a.ebscohost.com.proxy.libraries.uc.edu/ehost/search/advanced?vid=0%26sid=020904cf-79ab-4414-a7c2-75d3bb035837%40sessionmgr4008; then “Choose Databases” > Legal Collection	“TX ALL Text”
EBSCOhost	Wilson Omnifile	http://web.a.ebscohost.com.proxy.libraries.uc.edu/ehost/search/advanced?vid=0%26sid=020904cf-79ab-4414-a7c2-75d3bb035837%40sessionmgr4008; then “Choose Databases” > Wilson Omnifile	“TX ALL Text”
EBSCOhost	PsychINFO	http://web.a.ebscohost.com.proxy.libraries.uc.edu/ehost/search/advanced?vid=0%26sid=020904cf-79ab-4414-a7c2-75d3bb035837%40sessionmgr4008; then “Choose Databases” > PsychINFO	“TX ALL Text”
EBSCOhost	Social Work Abstracts	http://web.a.ebscohost.com.proxy.libraries.uc.edu/ehost/search/advanced?vid=0%26sid=020904cf-79ab-4414-a7c2-75d3bb035837%40sessionmgr4008; then “Choose Databases” > Social Work Abstracts	“TX ALL Text”
EBSCOhost	Women's Studies International	http://web.a.ebscohost.com.proxy.libraries.uc.edu/ehost/search/advanced?vid=0%26sid=020904cf-79ab-4414-a7c2-75d3bb035837%40sessionmgr4008; then “Choose Databases” > Women's Studies International	“TX ALL Text”
ProQuest	Criminal Justice Database	https://search-proquest-com.proxy.libraries.uc.edu/criminaljusticeperiodicals/advanced	“Anywhere”
ProQuest	PAIS	https://search-proquest-com.proxy.libraries.uc.edu/pais/advanced?accountid=2909	“Anywhere”
ProQuest	Dissertations & Theses Global	https://search-proquest-com.proxy.libraries.uc.edu/pqdtglobal/advanced?accountid=2909	“Anywhere”
Gale	Expanded Academic ASAP	https://go-gale-com.proxy.libraries.uc.edu/ps/start.do?p=EAIM%26u=ucinc_main	“Entire Document”
Gale	Opposing Viewpoints Resource Center	https://go-gale-com.proxy.libraries.uc.edu/ps/start.do?p=OVIC%26u=ucinc_main	N/A
FirstSearch	GPO Monthly Catalog	https://firstsearch-oclc-org.proxy.libraries.uc.edu/WebZ/FSPrefs?entityjsdetect=:javascript=true:screensize=large:sessionid=fsap05pxm1-1680-kekfmvf6-ue8qrf:entitypagenum=1:0	“Keyword”
FirstSearch	PapersFirst	https://firstsearch-oclc-org.proxy.libraries.uc.edu/WebZ/FSPrefs?entityjsdetect=:javascript=true:screensize=large:sessionid=fsap05pxm1-1680-kekfp1c9-afj7vm:entitypagenum=1:0	“Keyword”
ISI Web of Knowledge	Web of Science Core Collection	http://apps.webofknowledge.com.proxy.libraries.uc.edu/WOS_GeneralSearch_input.do?product=WOS%26search_mode=GeneralSearch%26SID=8Cuw8UrYzZEgCr5PC1f%26preferencesSaved=	“All Fields”
Office of Justice Programs	National Criminal Justice Reference Service	https://www.ncjrs.gov/App/search/AdvancedSearch.aspx	N/A
U.S. National Library of Medicine (NIH)	ClinicalTrials.gov	https://clinicaltrials.gov/ct2/search/advanced?cond=%26term=%26cntry=%26state=%26city=%26dist=	“Other terms”
Elsevier	Scopus	https://www-scopus-com.proxy.libraries.uc.edu/search/form.uri#basic	“All Fields”
–	Summon	http://uc.summon.ssc.proxy.libraries.uc.edu/advanced#!/advanced	“All Fields”
–	Nexis Uni	https://advance-lexis-com.proxy.libraries.uc.edu/practice/?pdmfid=1516831%26crid=a0cbd6ff-35ec-4447-892d-514653f7c08d%26config=00JAAzNzdlNTFkNS00MDk2LTQzMTItYWM4Mi1kZjM0NGVlNDNkNzEKAFBvZENhdGFsb2eMH3E8B5wUwBgbm9pcVO5K%26pdsearchterms=%26pdbcts=1598994468235%26pdqttype=and%26ecomp=qfJgkgk%26prid=8c98b919-11d2-43dc-8569-424a5069f689	“All Fields”
–	Science.gov	https://www.science.gov/scigov/desktop/en/search.html	“Full Record”
–	Google Scholar	https://scholar.google.com/	N/A

## APPENDIX C MeSH ANALYSIS RESULTS

**Table:**

**PMID**	7352849	15933353	4890477	9200635	6996635
**Title**	Therapeutic communities vs methadone maintenance. A prospective…	Cost utility analysis of co‐prescribed heroin compared with met…	Methadone treatment of randomly selected criminal addicts…	Dose‐related efficacy of levomethadyl acetate for treatment of…	Evaluation of heroin maintenance in controlled trial.
**Author Year**	Bale et al. (1980)	Dijkgraaf (2005)	Dole (1969)	Eissenberg (1997)	Hartnoll (1980)
**MeSH Headings**	Adult	Administration, Inhalation Adult	Adolescent Adult African Americans	Adult Analysis of Variance	Adolescent Adult
	Crime	Chronic Disease Cost of Illness Cost‐Benefit Analysis Crime/statistics & numerical data	Clinical Trials as Topic Crime*	Cocaine/urine	Clinical Trials as Topic Crime
		Drug Resistance Drug Therapy, Combination		Dose‐Response Relationship, Drug Double‐Blind Method	Dose‐Response Relationship, Drug
			Educational Status Ethnic Groups European Continental Ancestry Group		
	Female Follow‐Up Studies	Female	Follow‐Up Studies	Female	Female
	Heroin Dependence/psychology Heroin Dependence/rehabilitation* Humans	Health Resources/economics Health Resources/utilization Heroin/administration & dosage Heroin/economics* Heroin Dependence/economics* Heroin Dependence/rehabilitation Humans	Heroin* Humans	Heroin/urine Heroin Dependence/complications Heroin Dependence/drug therapy* Humans	Heroin/administration & dosage* Heroin Dependence/psychology Heroin Dependence/rehabilitation* Humans
		Injections, Intravenous			
	Male Methadone/therapeutic use*	Male Methadone/administration & dosage Methadone/economics* Multicenter Studies as Topic	Male Methadone/administration & dosage Methadone/therapeutic use* Morphine Dependence/drug therapy* Morphine Dependence/rehabilitation Motivation	Male Methadyl Acetate/administration & dosage* Middle Aged	Male Methadone/therapeutic use
		Narcotics/administration & dosage Narcotics/economics*	New York City	Narcotics/administration & dosage*	
					Outcome and Process Assessment (Health Care)
	Patient Acceptance of Health Care		Prisons		
		Quality‐Adjusted Life Years			
		Randomized Controlled Trials as Topic			Random Allocation
	Social Adjustment			Substance‐Related Disorders/complications	Social Adjustment
	Therapeutic Community*	Treatment Outcome		Treatment Outcome	
					United Kingdom
			Work		

**PMID**	12033654	18930603	16919749	90214	9651260
**Title**	A placebo‐controlled study of high dose buprenorphine in opiate…	Buprenorphine and methadone maintenance in jail and post‐releas…	Controlled trial of prescribed heroin in the treatment of opioi…	Double‐blind comparison of methadone and placebo maintenance tr…	Randomised trial of heroin maintenance programme for addicts wh…
**Author Year**	Krook (2002)	Magura et al. (2009)	March (2006)	Newman and Whitehill (1979)	Perneger (1998)
**MeSH Headings**	Adult	Adolescent Adult Aged	Adult	Adult Aged Ambulatory Care	Adult Ambulatory Care
	Buprenorphine/therapeutic use*	Buprenorphine/adverse effects Buprenorphine/therapeutic use*			
			Counseling/legislation & jurisprudence	Clinical Trials as Topic	
	Double‐Blind Method	Diagnosis, Dual (Psychiatry)	Drug Administration Schedule Drug Prescriptions/statistics & numerical data*	Double‐Blind Method	
	Female	Female Follow‐Up Studies	Female	Follow‐Up Studies	Female Follow‐Up Studies
	Humans	HIV Infections/complications HIV Infections/psychology Humans	HIV Seropositivity/epidemiology Health Status Hepatitis B/epidemiology Hepatitis C/epidemiology Heroin/therapeutic use* Humans	Heroin/urine Heroin Dependence/drug therapy* Hong Kong Humans	Health Status Heroin/administration & dosage* Heroin Dependence/psychology Heroin Dependence/rehabilitation* Humans
					Income Infusions, Intravenous Interpersonal Relations
	Male Middle Aged	Male Mental Disorders/complications Mental Disorders/psychology Methadone/adverse effects Methadone/therapeutic use* Middle Aged	Male Methadone/therapeutic use	Male Methadone/administration & dosage* Middle Aged	Male
	Narcotic Antagonists/therapeutic use*	Narcotics/adverse effects Narcotics/therapeutic use*	Narcotics/therapeutic use*	New York City	Narcotics/administration & dosage*
	Opioid‐Related Disorders/rehabilitation*	Opioid‐Related Disorders/rehabilitation*	Opioid‐Related Disorders/diagnosis Opioid‐Related Disorders/rehabilitation*		
	Patient Compliance	Patient Selection Prisons*		Patient Dropouts Placebos	
		Recurrence	Risk‐Taking		
		Socioeconomic Factors	Severity of Illness Index Social Support		Street Drugs Switzerland
	Treatment Outcome	Treatment Outcome		Time Factors	
		Young Adult			

## APPENDIX D PRELIMINARY SEARCH STRINGS

**Table:**

String no.	String
1	(“Medication assisted treat*” OR “Medication assisted therap*” OR drug* OR "therapeutic use*" OR opioid* OR “replacement therap*” OR “substitution therap*” OR pharmacotherap* OR “pharmacological treatment*” OR addict* OR agonist* OR “partial agonist*” OR methadone OR methadose OR dolophine OR buprenorphine OR suboxone OR sublocade OR naltrexone OR depade OR vivitrol OR revia OR “levomethadyl acetate” OR LAAM OR orlaam OR morphine OR analgesic* OR heroin OR narcotic OR heroin OR intravenous OR “dose‐response relationship*” OR “drug prescription*”)
2	(crim* OR incarcerat* OR convict* OR offend* OR offence* OR offense* OR reincarceration OR reconvict* OR reoffen* OR recidiv* OR rearrest* OR arrest* OR probation* OR parole* OR "community supervis*" OR “technical violat*” OR "drug court*" OR "special* court*" OR “treatment court*”)
3	#1 AND #2
4	(“Medication assisted treat*” OR “Medication assisted therap*” OR drug* OR "therapeutic use*" OR opioid* OR “replacement therap*” OR “substitution therap*” OR pharmacotherap* OR “pharmacological treatment*” OR addict* OR agonist* OR “partial agonist*” OR methadone OR methadose OR dolophine OR buprenorphine OR suboxone OR sublocade OR naltrexone OR depade OR vivitrol OR revia OR “levomethadyl acetate” OR LAAM OR orlaam OR morphine OR analgesic* OR heroin OR narcotic OR heroin OR intravenous OR “dose‐response relationship*” OR “drug prescription*”) AND (overdos*) AND (crim* OR “crim* history” OR incarcerat* OR convict* OR offend* OR offence* OR offense* OR reincarceration OR reconvict* OR reoffen* OR recidiv* OR rearrest* OR arrest* OR probation* OR parole* OR "community supervis*" OR “technical violat*” OR "drug court*" OR "special* court*" OR “treatment court*”)
5	(#3 OR #4) AND Date: From 1960 to Oct 31, 2020
